# Predicting the distribution of potentially suitable habitat in China for *Cirsium japonicum* Fisch. ex DC. under future climate scenarios based on the R‐optimized MaxEnt model

**DOI:** 10.1002/ece3.11653

**Published:** 2024-07-09

**Authors:** Hu‐Qiang Fang, Zi‐Xuan Jiang, Shi‐Mao Chen, Tao Xie, Yu Xue, Jia Song, Qing‐Shan Yang

**Affiliations:** ^1^ College of Pharmacy Anhui University of Chinese Medicine Hefei China; ^2^ Anhui Province Key Laboratory of Research & Development of Chinese Medicine Hefei China; ^3^ Institute of Conservation and Development of Traditional Chinese Medicine Resources, Anhui Academy of Chinese Medicine Hefei China

**Keywords:** *Cirsium japonicum* Fisch. ex DC., climate change, MaxEnt model, suitable area

## Abstract

*Cirsium japonicum* contains a variety of medicinal components with good clinical efficacy. With the rapid changes in global climate, it is increasingly important to study the distribution of species habitats and the factors influencing their adaptability. Utilizing the MaxEnt model, we forecasted the present and future distribution regions of suitable habitats for *C. japonicum* under various climate scenarios. The outcome showed that under the current climate, the total suitable area of *C. japonicum* is 2,303,624 km^2^ and the highly suitable area is 79,117 km^2^. The distribution of *C. japonicum* is significantly influenced by key environmental factors such as temperature annual range, precipitation of the driest month, and precipitation of the wettest month. In light of future climate change, the suitable habitat for *C. japonicum* is anticipated to progressively relocate toward the western and northern regions, leading to an expansion in the total suitable area. These findings offer valuable insights into the conservation, sustainable utilization, and standardized cultivation of wild *C. japonicum* resources.

## Graphical abstract

Utilizing the MaxEnt model, we forecasted the present and future distribution regions of suitable habitats for *Cirsium japonicum* Fisch. ex DC. under various climate scenarios. Our analysis focused on identifying the key environmental variables impacting habitat alterations and detailing the shifts in suitable habitats for *C. japonicum* across different timeframes and scenarios.

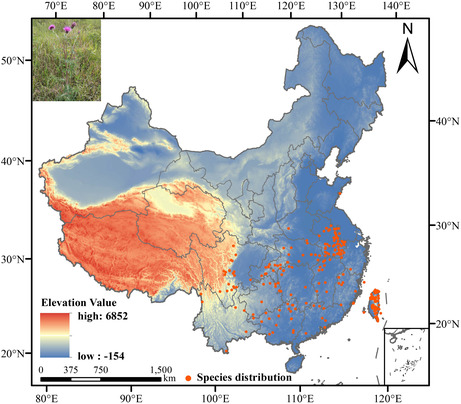

## 1 INTRODUCTION

The impact of climate change on humanity is extensive and far‐reaching, and it is a major concern for all countries in the world. Climate change is a series of environmental changes caused by the accumulation of greenhouse gases (GHG) in the atmosphere and the increasing temperature of the earth (Eckardt et al., 2023). The distribution and growth of plants are closely related to the environment in which they are found. The 5th Assessment Report of the United Nations Intergovernmental Panel on Climate Change (IPCC, 2014) states that by the end of the 21st century, the global average surface temperature will have risen by 0.3–4.8°C (IPCC, 2014; Vousdoukas et al., 2018). Climate change is likely to cause changes in the geographical distribution of creatures and loss of habitat in the coming period (Ranjitkar et al., 2016), which in turn will exacerbate the loss of good germplasm and accelerate the rate of species extinction (Beever et al., 2011; Bellard et al., 2012; Grinder & Wiens, 2023).

In recent years, various modeling methods such as the Maximum Entropy (MaxEnt) (Huang et al., 2023) Model, Maximum Likelihood (MaxLike) Model (Merow & Silander, 2014), and Generalized Additive Model (GAM) (Chan‐Espinoza et al., 2024), combined with spatial analysis techniques for geographic information, have been extensively utilized to forecast the influence of climate on species' potential habitats. Modern research has shown that the MaxEnt model performs better among the various species distribution models (Elith et al., 2006; Gao et al., 2020; Reddy et al., 2015). MaxEnt is an algorithm based on the theory of maximum entropy, which selects the predicted distribution with the highest entropy value from the eligible known distributions as the optimal solution of the model and establishes the interrelationships between the species distributions and the constraints (environmental factors) (Phillips et al., 2006). Using the MaxEnt model to speculate the potential habitats of creatures has the characteristics of small distortion and good stability (Zheng et al., 2021). It has a wide range of applications and plays an important role in the study of the impacts of climate change on species distribution and the management and conservation of endangered species, involving a wide range of disciplines such as agronomy (Ali et al., 2023), forestry (Yousaf et al., 2022), virology (Velu et al., 2023; Zakharova et al., 2020), etc.

*Cirsium japonicum*, a perennial plant belonging to the genus *Cirsium* within the Asteraceae family, is commonly found throughout various Asian countries, including China, Japan, Korea, and South Korea. In China, the distribution of *C. japonicum* is extremely wide, spreading over Sichuan, Fujian, and Taiwan, with the main distribution area extending from the Hengduan Mountains in the west to the Qinling‐Huaihe line in the north, i.e., it is mainly concentrated in the southern provinces, with a rapidly diminishing amount of resources to the north. *C. japonicum* is not only used as a traditional Chinese medicine but its roots and shoots are also edible. *C. japonicum* mainly contains flavonoids, polysaccharides, coumarins, and alkaloids, which have obvious pharmacological activities (Ma et al., 2019). Modern pharmacology has shown that *C. japonicum* has anti‐inflammatory, anti‐cancer, antihypertensive, hemostatic, and immune‐enhancing effects (Kim et al., 2008, 2010; Liu et al., 2006; Ma et al., 2018), and it has long been used to treat hepatitis, osteoporosis, hypertension, and uterine and liver cancers (Kim et al., 2015; Liu et al., 2006).

At present, the supply of *C. japonicum* mainly relies on wild harvesting, and with the increasing market demand, its wild resources are becoming more and more scarce, which has become a bottleneck for the sustainable development of herb‐related industries. The selection of the planting area of *C. japonicum* is the key to its industrialization, and there are no studies in the literature on the response of suitable areas of *C. japonicum* to climate change. so this study aims to establish an ecologically suitable distribution model of *C. japonicum* and study the trend of its ecologically suitable area and the dominant environmental impact factors in the future and under different greenhouse gas (GHG) emission concentrations, which is of profound significance for the rational utilization of the existing resources and standardized planting of the *C. japonicum*.

## 2 MATERIALS AND METHODS

### 2.1 Data collection

In this study, we collected information on the coordinates of specimens of 714 *C. japonicum* within China, of which 56 came from the database of the 4th National Census of Traditional Chinese Medicine Resources, and the other data were collected from the Chinese Virtual Herbarium (http://www.cvh.ac.cn/; accessed on September 23, 2023) and the Global Biodiversity Information Facility database (https://www.gbif.org/; accessed on September 29, 2023). For sample points where only the address of the collection point was recorded, we used Google Earth (http://ditu.google.cn/) to query their latitude and longitude (Ji et al., 2021). We converted the latitude and longitude information of these loci into degrees, minutes, and seconds and saved them in .csv format.

Bioclimatic variables have a vital effect on shaping the patterns of suitable habitats for species. Bioclimatic variables characterize changes in rainfall and temperature values over some time in natural climates, and they are also suitable for intercontinental, global, and other large‐scale range projections (Choudhury et al., 2016; Zhang et al., 2016). In this experiment, 2.5 min resolution climate data were used, all derived from Worldclimate (http://www.worldclim.org/; accessed on November 25, 2023), which is now widely used to run species distribution models that reflect temperature and precipitation, etc., at the study area scale. Included in this database are 19 bioclimatic variables for the contemporary period (1960–1990), and a total of 6 future bioclimatic variables for different periods and scenarios in the 2050s (average for 2041–2060) and 2070s (average for 2061–2080) (CMIP5).

Representative Concentration Pathways (RCPs) are a set of comprehensive scenarios that integrate greenhouse gas (GHG) concentrations and emissions (IPCC, 2014). These scenarios, featured by the IPCC in the Fifth Assessment Report, serve as models for GHG emissions at various concentration levels in the 21st century, considering human activities' impact on climate change (Moss et al., 2010). RCPs encompass RCP2.6, RCP4.5, RCP6.0, and RCP8.5, each reflecting distinct GHG emission levels – low, medium, and high, respectively (Remya et al., 2015). RCP4.5 and RCP6.0 fall under medium GHG emission scenarios, with RCP4.5 being the preferred option. Therefore, for modeling the potential suitable distribution of *C. japonicum* in the future, we selected RCP2.6 (low GHG emissions), RCP4.5 (medium GHG emissions), and RCP8.5 (high GHG emissions) scenarios.

### 2.2 Data processing

#### 2.2.1 Analysis and processing of occurrence data

**FIGURE 1:**
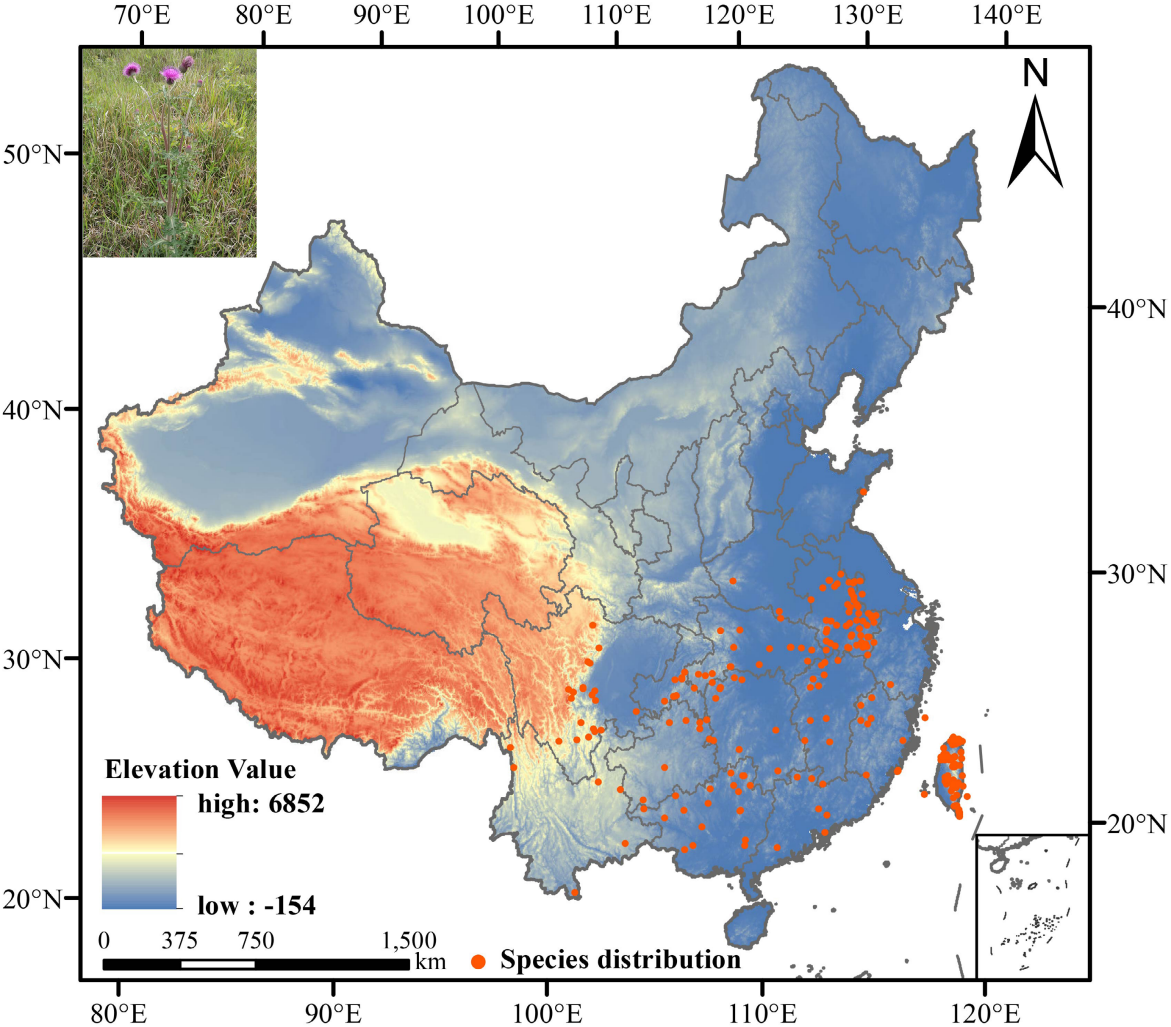
Distribution of the sample points of *C. japonicum* in China.

Species sampling points are usually concentrated in a certain geographic area, and the use of these dense point data will cause spatial autocorrelation, which will lead to overfitting of MaxEnt model results (Ji et al., 2020). To minimize the experimental error, we divided the study area within China into several grids, each of which is a size of about 4.8 × 4.8 km. In each grid, we selected only one distribution point (Rodríguez‐Castañeda et al., 2012). According to the resolution of the bioclimatic data, we used the ENMtool tool to remove duplicate points and sampling points with similar spatial distribution, we finally obtained 254 valid sample information, and we finally obtained 254 valid sample information. Based on the sample point coordinate information obtained from the above processing, we saved it as a .csv format file following the input format of MaxEnt software and plotted the distribution of sample points of *C. japonicum* within China (Figure 1).

#### 2.2.2 Analysis and processing of environmental variables

**FIGURE 2:**
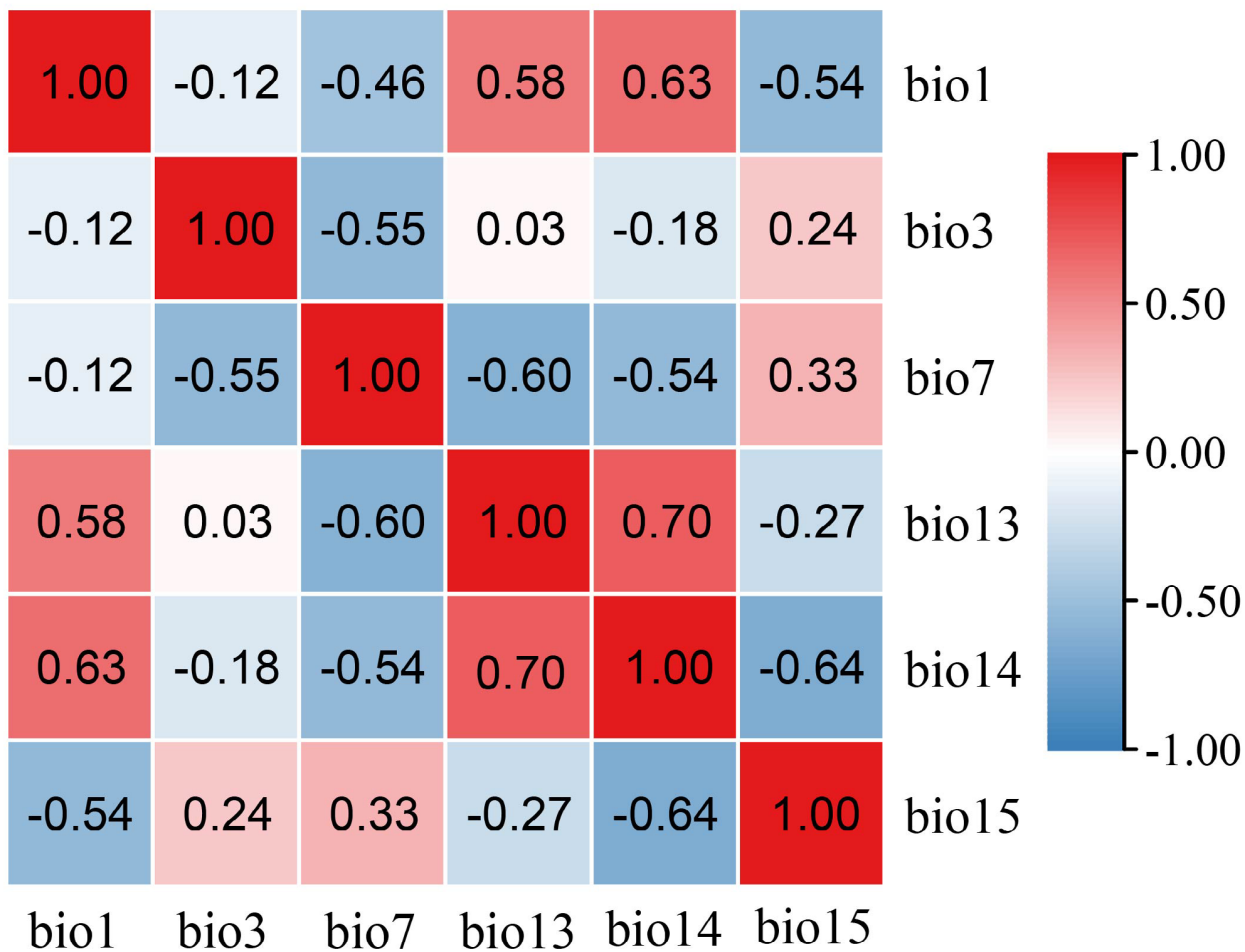
Heat map displays the Pearson correlation among the nine environmental variables utilized in MaxEnt modeling. Detailed descriptions of these environmental variables can be found in Table 1.

The 19 climatic data represent environmental variables such as temperature and precipitation in annual, quarterly, and monthly intervals, which are correlated with each other (Thapa et al., 2018). Therefore, before modeling, we need to consider whether these variables have strong correlations with each other and screen the variables with strong correlations to reduce the errors caused by environmental variables. We imported the pre‐screened sample point data and 19 current environmental variables into MaxEnt modeling software (www.cs.princeton.edu/~schapire/MaxEnt) for pre‐experimentation and 3/4 of the sample distribution data were set as the training set, and the remaining 1/4 as the test set for pre‐experimentation, other parameters remained unchanged, and run 1 time to get the contribution value data of 19 climate factors. At the same time, we imported the above sample point data and 19 environmental data into ArcGIS (http://www.arcgis.com) and resampled them to obtain the values of 19 environmental variables corresponding to each sample point, and used SPSS20 (https://www.ibm.com/cn‐zh) to perform Pearson correlation analysis on these environmental variable values to derive the correlation coefficients. We stipulated that when the correlation coefficient of a pair of environmental variables |*R*| ≥ .8, the one with low contribution among the two correlated variables was excluded based on the results of the pre‐experiments (Thapa et al., 2018). We finally screened out 6 environmental variables from 19 environmental variables to participate in the model prediction (Figure 2).

### 2.3 Model parameter optimization and modeling

The current study shows that when MaxEnt is run with default parameter settings, it will likely result in poor model prediction performance. The performance and accuracy of the model will vary depending on the adjustment of the regularization multiplier (RM) and feature combination (FC). For the tuning of these two parameters we mainly used R v3.6.3 to mobilize the kuenm package (Cobos et al., 2019) to generate different model combinations for model optimization, and then based on the optimization results, we tuned the FC and RM parameters in MaxEnt to ensure that the model runs with the best prediction performance.

We divided the distribution loci data obtained from the pre‐screening into four parts, three of which were used for the training set and one for the test set. FC consists of five combinations of feature classes l (linear), q (quadratic), p (product), t (threshold), and h (hinge), including 29 types of combinations such as l, lq, qp, pth, lqpth, etc. The RM constants typically range from 0.1 to 4.0, incrementing by 0.1 for a total of 40 values. kuenm package can run automatically, combining different values of RM and different combinations of FC to form 1160 kinds of models and analyze them to derive their performance parameters. The optimal model is selected based on the best‐performing parameters, ensuring that it meets specific criteria: (1) statistically significant results and (2) an omission rate of less than or equal to 5%, and (3) ΔAICc is 0 (i.e., AICc is minimized). Finally, the model that meets the above three requirements is the best model we choose (Huercha et al., 2020; Liu & Shi, 2020).

We imported the sample point distribution data in .csv format and the six environmental variables (including contemporary and future climate data) in asc format into the MaxEnt model, set the FC and RM values according to the parameters of the optimal model, and also set 3/4 of the sample point distribution data as the training set and the remaining 1/4 as the test set, and ran the model setup for 10 repetitions (Wu et al., 2021). Finally, we proceed to the next step of analysis from the resulting data obtained from the model run.

### 2.4 Evaluation of the model

The receiver operating characteristic (ROC) curve's area under the curve (AUC) was employed to assess the model's predictive capability. AUC is a commonly utilized metric in evaluating SDM models, unaffected by threshold adjustments, making it a reliable indicator of the model's predictive accuracy. The accuracy of model prediction is positively correlated with the size of AUC, which usually ranges from 0.5 to 1. 0.5 ≤ AUC < 0.6 results in invalid model prediction, 0.6 ≤ AUC < 0.7 results in poor model performance, 0.7 ≤ AUC < 0.8 results in fair model performance, 0.8 ≤ AUC < 0.9 results in fine model performance, and 0.9 ≤ AUC < 1 results in outstanding model performance (Xu et al., 2023). The AICc value in the above model optimization results is an index for evaluating the complexity of the model, and AUC is an index for evaluating the accuracy of the model, and both can be used to evaluate the predictive performance of the model.

### 2.5 Potential habitat evaluations

Based on the final prediction results from MaxEnt, the output format was ASCII raster layer, which was imported into ArcGIS v 10.4 and manually classified the prediction results of *C. japonicum*'s suitable areas into suitable classes using the reclassification tool. P denotes the suitability index of the distribution of *C. japonicum*, which ranges from 0 (least suitable) to 1 (most suitable). We used the maximum test sensitivity plus specificity threshold (MTSPS) as the dividing line between suitable and non‐suitable areas, and based on this, we categorized the suitable areas into 3 classes. Hence, the classification outcomes were as follows: *p* < MTSPS (unsuitable area); MTSPS ≤ *p* < .35 (low‐suitability area); .35 ≤ *p* < .55 (moderate‐suitability area); and .55 ≤ *p* (high‐suitability area).

### 2.6 Changes in suitable habitat area and centroids in the future

To delve deeper into the evolving patterns of suitable habitats for *C. japonicum* under various future scenarios, we employed the SDMtoolbox within the ArcGIS toolkit. This enabled us to compute the total area of suitable habitats, highly suitable areas, and the centroid migration direction and distance across different scenarios in both current and future time frames. We also mapped and analyzed the changes in geographic distribution patterns, center‐of‐mass migration paths, and migration distances of *C. japonicum* under current and future scenarios. Changes in the geographic distribution pattern of the habitat reflect changes in the area of suitable areas, and changes in the center of mass reflect the direction of migration of suitable area locations.

## 3 RESULTS

### 3.1 Model parameters and performance evaluation

**FIGURE 3:**
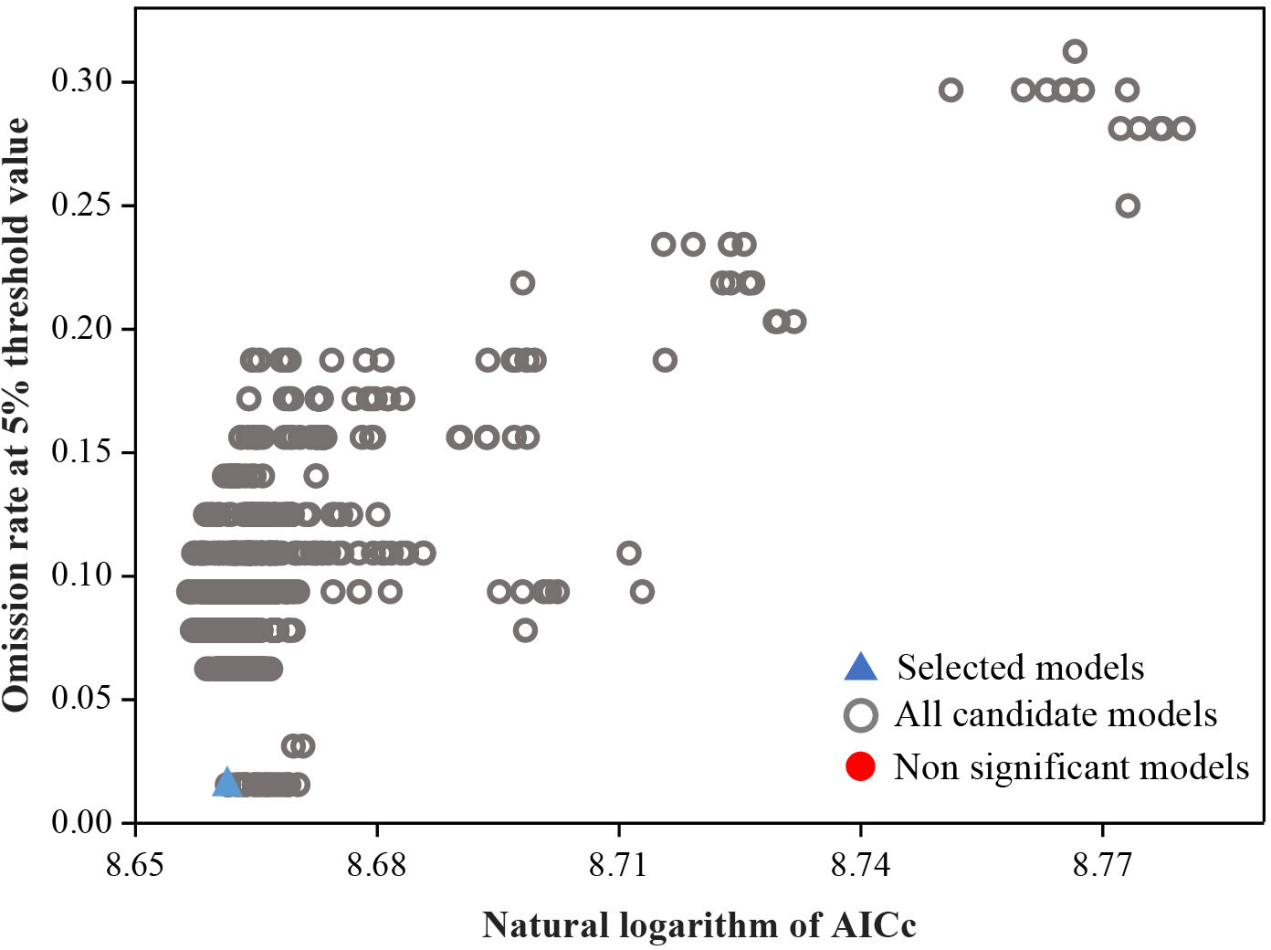
MaxEnt model parameter optimization results.

While forecasting the potential distribution areas of *C. japonicum* in various time frames and scenarios in China using the MaxEnt model, we observed a ΔAICc of 17.2379 and an omission rate of 0.0781 with the default settings (FC = lqpth; RM = 1). However, with optimized parameters (F = t; RM = 2.2), the ΔAICc dropped to 0, and the omission rate reduced to 0.0156. It is worth noting that the AUC value of the model is as high as 0.922 at this time. This indicates that the optimized model has high accuracy and low overfitting AUC value is as high as 0.933, which indicates that the model is optimized for high prediction accuracy and low overfitting (The results of the model optimization are shown in Figure 3). Therefore, it is more accurate to first use model optimization to derive the best model and then project the suitable distribution of *C. japonicum* under various climate conditions than to run the model with the default parameter set.

### 3.2 Influencing of major environmental factors

**FIGURE 4:**
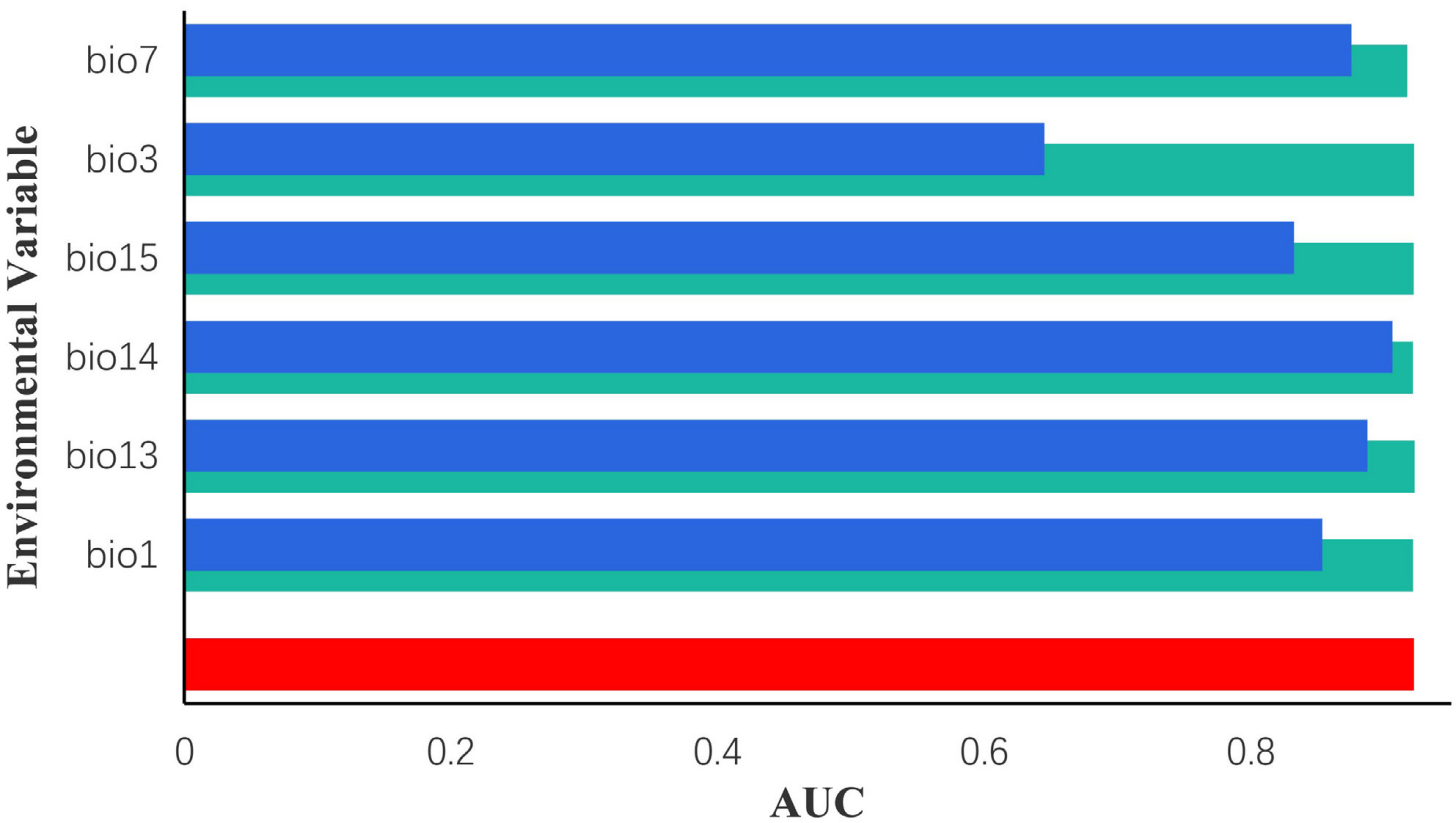
The results of the Jackknife testing for the environmental variables utilized in the modeling process. Detailed explanations of these environmental variables can be found in Table 1.

At the end of the successful establishment and operation of the MaxEnt model, we applied the jackknife method to evaluate the significance of each environmental factor using the result dataset obtained. According to the results, it can be seen that the highest contribution of each environmental variable used to construct the model is mainly bio7 (41.7%), bio14 (40.5%), bio13 (15.3%), and the cumulative contribution of the three is as high as 97.5% (Table 1). Based on the results of the jackknife test for the materiality of the environmental factors, it can be seen that the bio7, bio14, and bio13 environmental variables have the largest normalized AUC values when the model is run using a sole environmental factor (Figure 4). Thus the three variables bio7, bio14, and bio13 were the key environmental factors that influenced the suitable distribution of *C. japonicum*.

**TABLE 1:** Percentage contribution and permutation importance of environmental variables of *C. lineare* in the fitted MaxEnt model.

Symbol	Environmental variable	Percentage contribution (%)	Permutation importance
bio7	Temperature annual range	41.7	49.7
bio14	Precipitation of driest month	40.5	20.9
bio13	Precipitation of wettest month	15.3	14.0
bio1	Annual mean temperature	2.1	8.1
bio15	Precipitation seasonality	0.4	7.2
bio3	Isothermality	0	0.1

### 3.3 Suitable area for the current period

**FIGURE 5:**
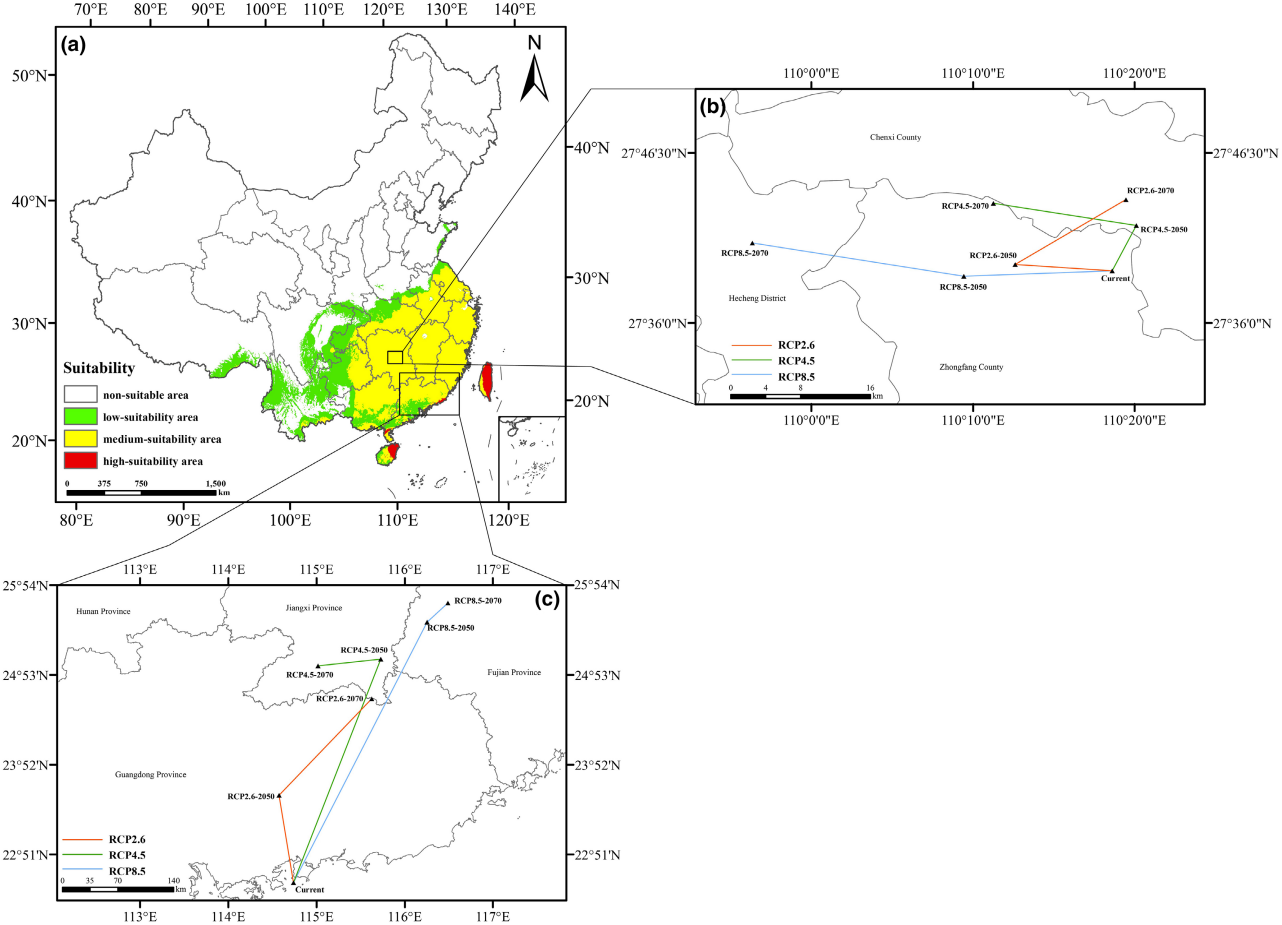
The current potential habitat distribution and centroid migration. (a) showcases the current potential distribution area, (b) and (c) display the centroid migration under three distinct climate scenarios.

Based on the results of the MaxEnt model run, combined with the above method of dividing the suitability classes, we mapped the current potential distribution of *C. japonicum* (Figure 5a). The white area is the non‐suitable area (0.00 ~ MTSPS); the green area represents the low suitable area (MTSPS ~ 0.35); the yellow area is the medium suitable area (0.35–0.55); and the red area represents the high suitable area (0.55–1.00). From the potential distribution map of *C. japonicum* within China, the total suitable potential distribution area of *C. japonicum* in the current period (including low, medium, and high suitability areas) is about 2,303,624.424 km^2^, which is about 23.9961% of the total area of the country, and it is mainly located in the middle and south of East China, Central China, South China, and the southeastern part of Southwest China. The total area of the highly suitable area for *C. japonicum* is about 79,116.9290 km^2^, accounting for about 0.8241% of the total area of the country. It is mainly located in the eastern part of Hainan Province, the east‐central part of Taiwan Province, and the coastal area of Guangdong Province.

### 3.4 Suitable area for future periods

**FIGURE 6:**
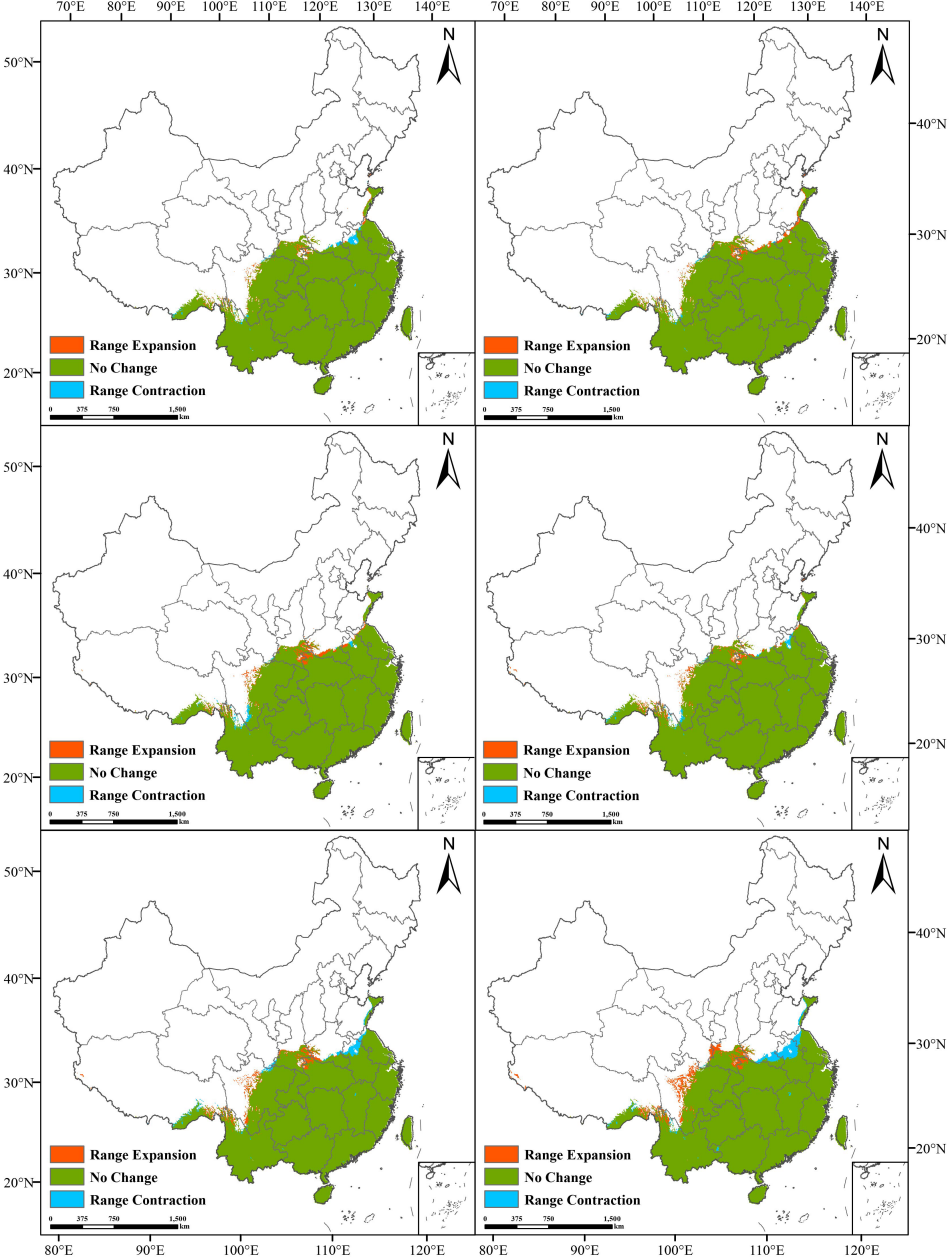
Changes in spatial and geographical patterns of total suitable habitat in the 2050s and 2070s compared to the present (red indicates expansion, green indicates stability, and blue indicates contraction).

During this study, we forecasted the potential distribution of suitable habitats for *C. japonicum* in China across six distinct scenarios. The findings indicated that, despite future climate changes, the overall location of suitable areas for *C. japonicum* would remain relatively stable, predominantly spanning East China, Central China, the eastern portion of Southwest China, and South China (Figure 6). The total area of suitable areas under the three different scenarios increased by about 0.5264% (RCP2.6), 1.8515% (RCP4.5), and 0.8250% (RCP8.5) compared with the current scenario for the period of 2041–2060. for the period of 2061–2080, the increase over the current scenario was respectively 1.9372% (RCP2.6), 1.3942% (RCP4.5), and 1.1453% (RCP8.5). From the data, it is clear that the total suitable area of *C. japonicum* tends to expand in the future. In particular, under the RCP2.6 GHG emission concentration scenario in the 2070s, the area of the suitable zone will reach its maximum, which is 2,348,250.0450 km^2^.

**FIGURE 7:**
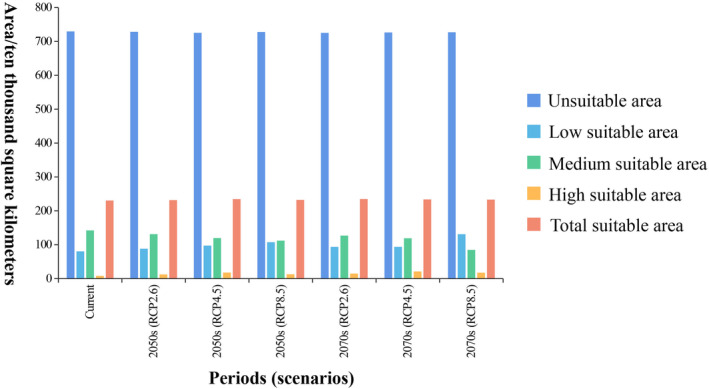
The variations in the area of suitable and unsuitable zones across different periods and scenarios.

Relative to the change in the total suitable area, the percentage of highly suitable areas changes significantly (Figure 7). At the same concentration of GHG emissions: under the RCP2.6 scenario, the area of the highly suitable zone increases over time from the current level of 79,116.9290 km^2^, first to 123,690.5789 km^2^, and then to 147,164.0708 km^2^. The total area of the highly suitable zone under the RCP4.5 scenario increases over time first to 178,104.0391 km^2^ and then to 208,558.9463 km^2^. Under the RCP8.5 scenario, the total area of the highly suitable area increases from the current to 128,541.1896 km^2^ and then to 172,941.6032 km^2^.

From the same period, as the concentration of GHG emissions increased, the area share of highly suitable areas first increased from 123,690.5787 km^2^ to 178,104.0391 km^2^ in the 2050s and then decreased to 128,541.1896 km^2^ in the 2070s. In the 2070s, the area share of highly suitable areas increased from 147,164.0708 km^2^, and increased to 208,558.9463 km^2^, and then decreased to 172,941.6032 km^2^. Generally speaking, within a certain range, with the increase of GHG emission concentrations during the same period, the area of highly suitable areas shows a trend of increasing and then decreasing. Figure 6 illustrates the variations in the area of suitable and unsuitable zones across different periods and scenarios.

### 3.5 Centroids migration of suitable areas in the coming period

To investigate the prospective alterations in the spatial distribution of suitable habitats for *C. japonicum*, we first installed the SDMtoolbox and employed the Distribution Change tool. This tool enabled us to analyze the magnitude and orientation of variations in both total suitable areas and regions with high suitability, producing trajectory maps illustrating changes in the center of mass. When we used the MTSPS value (MTSPS = 0.1578) as the threshold to study the total suitable areas change, each center of mass fell in Huaihua City, Hunan Province (Figure 5b). At this time the current center of mass was located in Zhongfang County, Huaihua City (110.31°E, 27.6535°N). From the current period to the 50s of the 21st century, the center of mass in the scenarios of RCP2.6, RCP4.5, and RCP8.5 moves 9.8718 km, 5.7633 km, and 15.0719 km to the northwest, northeast, and southwest directions, respectively, at different angles. From the 2050s to the 2070s, the center of mass in the scenarios of RCP4.5 and RCP8.5 moves 14.7770 km and 21.7878 km to the northwest for the RCP4.5 and RCP8.5 scenarios, respectively, while the center of mass shifts 13.4509 km to the northeast for the RCP2.6 scenario. In general, the total suitable area for *C. japonicum* ultimately expands to the west and north for all GHG emission scenarios (figure 7).

**FIGURE 8:**
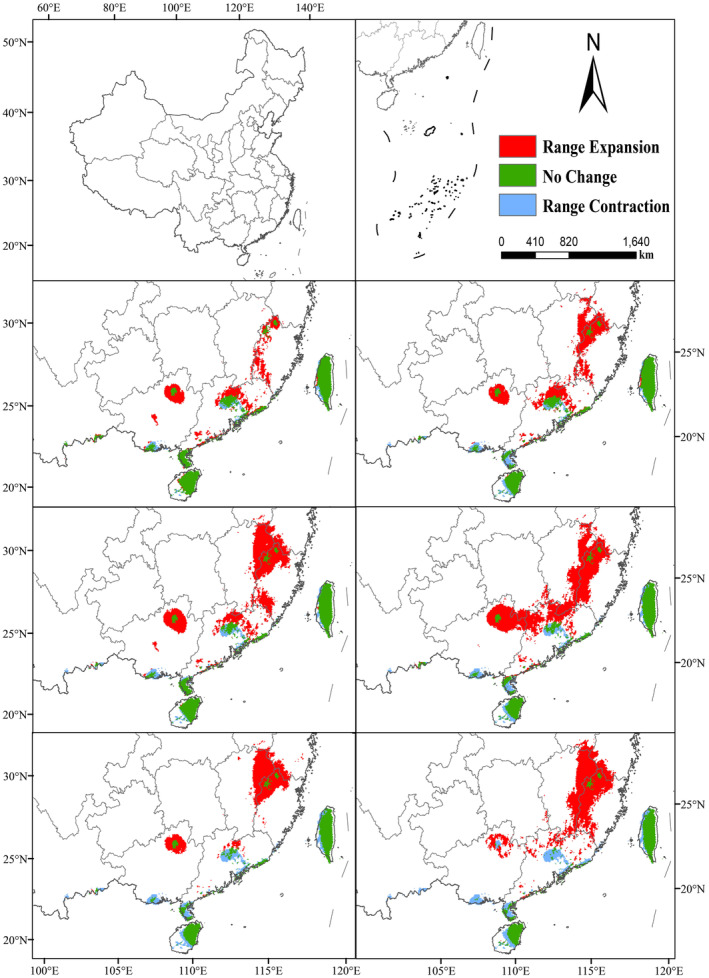
Spatial and geographical pattern of change in the highly suitable habitat for *C. japonicum* in the 2050s and 2070s compared with the current (red indicates expansion area, green indicates stable area, and blue indicates contraction area).

In addition, we also investigated the changes in the highly suitable area when the threshold was set at 0.55 (Figure 5c). At this time, the current center of mass is located in the coastal Guangdong Province (114.737°E, 22.5314°N), and the RCP4.5 and RCP8.5 centers of mass have a wide range of variation, and the migration distance increases with the increase of GHG concentration. From 2050s, the centers of mass migrated 111.1513 km, 298.9564 km, and 362.0363 km for the three scenarios of RCP2.6, RCP4.5, and RCP8.5, respectively, with the center of mass RCP2.6‐2050s located in Guangdong Province (115.625°E, 24.6155°N), and the center of mass RCP4.5‐2050s and RCP8.5‐2050s is located in Jiangxi Province (115.017°E, 24.9862°N) and Fujian Province (116.489°E, 25.6982°N), respectively, and the centers of mass in the three scenarios move by 161.6533 km, 72.1167 km, and 33.9109 km, respectively, from the 2050s to the 2070s, 33.9109 km. Similarly, the highly suitable area for *C. japonicum* will also expand in the west and north (Figure 8).

### 3.6 Cultivation zones in production areas

The formation and development of *C. japonicum* cultivation areas are closely related to the characteristics of natural resources, ecological and environmental factors, socio‐economic development, and scientific and technological progress. The following principles should be taken into consideration when dividing China's *C. japonicum*‐producing areas into cultivation zones: first, the authenticity of *C. japonicum* herbs; second, the ecological adaptability of *C. japonicum*; third, the proportion of *C. japonicum* cultivation in each ecological region and its development prospects; and fourth, the changes in global climatic and environmental conditions and the development of cultivation technology. According to the above principles, combined with the results of the distribution of the ecological adaptability of *C. japonicum*, the cultivation zones of *C. japonicum*‐producing areas are divided as follows.

The most suitable area: It is mainly located in the southeastern areas of Guangxi, Guangdong, Hainan, Taiwan, and other provinces. The area has sufficient sunshine, abundant rainfall, an average annual temperature of 15–24°C, precipitation of 1100–1900 mm, and a frost‐free period of more than 300 days. Suitable area: Mainly located in East China and some parts of Central China, including Hunan, Fujian, Jiangxi, the whole territory of Zhejiang, as well as the southern part of Anhui, the southern part of Jiangsu, the southern part of Hubei, the eastern part of Guizhou and other parts of the region. The area has sufficient sunshine, abundant rainfall, an average annual temperature of 13–20°C, precipitation of 580–2100 mm, frost‐free period of more than 200 days. Sub‐suitable areas: southeastern Tibet, all of Yunnan, eastern Sichuan, western Guizhou, western Chongqing, northern Hubei, and other areas; unsuitable areas: areas where *C. japonicum* basically cannot grow, i.e., areas other than the most suitable areas, suitable areas, and sub‐suitable areas.

## 4 DISCUSSION

*Cirsium japonicum* is a genus of *Cirsium* in the family Asteraceae, which is mainly distributed in China, Japan, and other Asian countries. This study is the first to predict the potential distribution of *C. japonicum* in China. We collected distribution data of *C. japonicum* from GBIF, CVH, and the Fourth National Census of Traditional Chinese Medicinal Resources (Anhui Province), and utilized the MaxEnt model to predict the distribution of *C. japonicum* and its dominant environmental factors under different scenarios of the current and future periods in China.

### 4.1 The precision of the experiment versus the accuracy of the results

MaxEnt is a model that has been widely used in recent years with stable and accurate predictions (Yang et al., 2024). However, studies have shown that unoptimized MaxEnt models may lead to inaccurate predictions. For example, early studies have shown that most (87%) of the MaxEnt model experiments used data affected by sampling bias (Yackulic et al., 2013), resulting in overfitting of the experiment and false positive results (Araújo & Guisan, 2006; Kadmon et al., 2004). Therefore, before we ran the MaxEnt model, we screened the sample point distribution data and optimized the model parameters, and the optimized model ΔAICc = 0 and AUC = 0.922, which indicated that the predictive performance of the optimized model was outstanding and the predictive results obtained from the experiments were reliable.

### 4.2 The influence of the dominant environmental factor on *C. japonicum*

As a medicinal and edible perennial herb, *C. japonicum* is affected by the environment in which it lives at all stages of growth, development, reproduction, and genetics (Ma & Sun, 2018).

According to the results of the model, we analyzed the results of the Jackknife test and the ranking of the contribution rate. bio7 had the greatest influence among the environmental variables affecting the distribution of *C. japonicum* in different periods and scenarios, with a contribution rate as high as 97.5%. bio7, bio14, and bio13 were the top three dominant environmental factors. Hence, the distribution of *C. japonicum* was mainly determined by temperature annual range, precipitation of the driest month, and precipitation of the wettest month, which suggests that the growth of *C. japonicum* is sensitive to temperature range and rainfall range. With future climate change, *C. japonicum* may be challenged to adapt to hotter conditions. Habitats located in the southern region will be rated less suitable, and *C. japonicum* populations may migrate to higher elevations or higher latitudes. Precipitation is another key factor affecting their growth. Climate change may lead to changes in regional precipitation patterns, with the driest month being the lower limit and the wettest month the upper limit of *C. japonicum*'s survival requirements, and overly arid or flooded environments forcing *C. japonicum* populations to seek out sites with more favorable water availability.

### 4.3 Changes in potential geographic distribution under climate change scenarios

Based on the experimental results, we can see that from the current period to the 2070s, the distribution location of the suitable area of *C. japonicum* has not changed much, and it is mainly located in East China, Central China, East Southwest China, and South China, etc. As time progressed, the total suitable area increased under all GHG emission concentration scenarios relative to the current period, with the largest increase in total area to 2,348,250 km^2^ under the RCP2.6 scenario in the 2070s. In addition, there is a certain pattern between the area of highly suitable areas and the concentration of GHG emissions, in the same period, the greater the concentration of GHG emissions, the greater the area of highly suitable areas, of which the maximum increase in the area of highly suitable areas can be up to 208,559 km^2^ under the scenario of RCP4.5 in the 2070s. In the course of global GHG emissions, the greenhouse effect will gradually intensify, and the Chinese climate will transition to a warmer and more humid climate (Yan et al., 2021), which is very suitable for *C. japonicum* growth at this time. As with other plants in the genus *Cirsium* (Fang et al., 2024), the total area of suitable habitat for *C. japonicum* increases over time, suggesting that the population dominance of *C. japonicum* is increasing in future periods.

In addition, the location of the center of gravity will also show a certain pattern of change with the changing time and GHG emission scenarios. At present, the center of mass of the total suitable area of *C. japonicum* is located in Zhongfang County, Huaihua City, Hunan Province (110.31°E, 27.6535°N), and the center of mass of the highly suitable area is located in the coast of Guangdong Province (114.737°E, 22.5314°N). The centers of mass in different periods and under different GHG emission scenarios are located to the north or west of the current center of mass, i.e., there is a tendency for the center of mass to move to higher altitudes or higher latitudes. From the projected results, the change in the location of the total suitable area is almost the same as the change in the center of mass with the change in the future climate, and it will be extended to the western and northern regions. This corroborates with previous findings that most species will gradually move to higher altitudes and latitudes to adapt to new natural environments as global warming increases (Lamprecht et al., 2018; Li et al., 2020; Wu et al., 2021).

### 4.4 Conservation and cultivation management

The rapid decline of wild resources of *C. japonicum* is due to the interaction of intrinsic and external factors, including its medicinal value and growth cycle, external disturbances, and climate change. Therefore, a multifaceted approach to its conservation is needed. (1) Strengthening artificial selection and promoting cultivation to supplement medicinal use. Artificial cultivation areas can be determined based on the most suitable areas predicted by our model, especially in highly suitable habitats. (2) Enhance in situ conservation by establishing protected areas through existing natural populations (Xie et al., 2022). As a medicinal plant, *C. japonicum* has important medicinal and economic values. With the increasing market demand, *C. japonicum* is often dug by local farmers and its wild resources are becoming more and more scarce. It was also found during the field trip that the inflorescences of *Cirsium* plants such as *C. japonicum* and *C. lineare* are susceptible to insect damage. Therefore, appropriate human intervention is an important means to promote the recovery and expansion of the resources; (3) Raise awareness of the importance of climate change, medicinal plant resources, and ecological protection.

However, we applied the MaxEnt model to analyze the relationship between the potential suitable distribution of *C. japonicum* and environmental variables, and the results we obtained are only theoretical speculations. Secondly, the six environmental variables we applied are not complete substitutes for all influencing factors. Asteraceae usually have crown hairs, which can rely on wind to drive seed dispersal (Panero & Crozier, 2016), so wind speed and direction also affect the extent of their distribution. In addition, solar radiation, soil type and land use changes (Xu et al., 2018), and human activities (Lamprecht et al., 2018; Liu et al., 2024; Xu et al., 2024; Zangiabadi et al., 2021) all have a non‐negligible effect on the distribution of species. According to the field study, it was found that the wild resources of *C. japonicum* have been drastically reduced due to over‐excavation, deforestation, and misuse of fertilizers and pesticides. It was also found during the field trip that Cirsium inflorescences such as *C. japonicum* and *C. lineare* are susceptible to insect infestation. Thus, employing a diverse array of environmental variables in habitat suitability modeling can enhance the accuracy and realism of the model, raising its overall standard of precision (Xie et al., 2023).

## 5 CONCLUSION

In this study, the parameters of the MaxEnt model were adjusted and optimized by using R to comprehensively analyze the distribution characteristics and change trends of *C. japonicum* in China based on the loci data of *C. japonicum* in China and the environmental data of current and future periods. The results showed that the model fit was excellent. Among the 19 environmental variables, bio7 (temperature annual range) stood out as the foremost factor affecting the potential suitability of *C. japonicum*. Relative to the current period, due to the impact of future climate, the suitability distribution area of *C. japonicum* will be shifted to the higher altitude or higher latitude areas in the west and north and gradually expanded. With the ongoing trend of global warming, the findings of this study could serve as a valuable reference for devising efficient schemes for adapting to climate change and conserving biodiversity.

## AUTHOR CONTRIBUTIONS

**Hu‐Qiang Fang:** Formal analysis (equal); methodology (equal); writing – original draft (equal). **Zi‐Xuan Jiang:** Validation (equal). **Shi‐Mao Chen:** Validation (equal). **Tao Xie:** Visualization (equal). **Yu Xue:** Formal analysis (equal). **Jia Song:** Validation (equal). **Qing‐Shan Yang:** Conceptualization (equal); supervision (equal).

## CONFLICT OF INTEREST STATEMENT

The authors declare no conflict of interest.

## Data Availability

Geographical data and maps are available via ZENODO http://doi.org/10.5281/zenodo.10951710.
